# Geometric and mechanical evaluation of 3D-printing materials for skull base anatomical education and endoscopic surgery simulation – A first step to create reliable customized simulators

**DOI:** 10.1371/journal.pone.0189486

**Published:** 2017-12-18

**Authors:** Valentin Favier, Nabil Zemiti, Oscar Caravaca Mora, Gérard Subsol, Guillaume Captier, Renaud Lebrun, Louis Crampette, Michel Mondain, Benjamin Gilles

**Affiliations:** 1 Montpellier Laboratory of Informatics, Robotics and Microelectonics (LIRMM), ICAR team, French National Centre for Scientific Research (CNRS), Montpellier University, Montpellier, France; 2 ENT department, University Hospital of Montpellier, Gui de Chauliac Hospital, Montpellier, France; 3 LIRMM, DEXTER team, CNRS, Montpellier University, Montpellier, France; 4 Anatomy laboratory, School of Medicine, Montpellier University, Montpellier, France; 5 Evolutionary Sciences Institute of Montpellier, MRI-ISEM, University of Montpellier, Montpellier, France; 6 AnatoScope SA, Montpellier, France; Universita degli Studi di Napoli Federico II, ITALY

## Abstract

**Introduction:**

Endoscopic skull base surgery allows minimal invasive therapy through the nostrils to treat infectious or tumorous diseases. Surgical and anatomical education in this field is limited by the lack of validated training models in terms of geometric and mechanical accuracy. We choose to evaluate several consumer-grade materials to create a patient-specific 3D-printed skull base model for anatomical learning and surgical training.

**Methods:**

Four 3D-printed consumer-grade materials were compared to human cadaver bone: calcium sulfate hemihydrate (named Multicolor), polyamide, resin and polycarbonate. We compared the geometric accuracy, forces required to break thin walls of materials and forces required during drilling.

**Results:**

All materials had an acceptable global geometric accuracy (from 0.083mm to 0.203mm of global error). Local accuracy was better in polycarbonate (0.09mm) and polyamide (0.15mm) than in Multicolor (0.90mm) and resin (0.86mm). Resin and polyamide thin walls were not broken at 200N. Forces needed to break Multicolor thin walls were 1.6–3.5 times higher than in bone. For polycarbonate, forces applied were 1.6–2.5 times higher. Polycarbonate had a mode of fracture similar to the cadaver bone. Forces applied on materials during drilling followed a normal distribution except for the polyamide which was melted. Energy spent during drilling was respectively 1.6 and 2.6 times higher on bone than on PC and Multicolor.

**Conclusion:**

Polycarbonate is a good substitute of human cadaver bone for skull base surgery simulation. Thanks to short lead times and reasonable production costs, patient-specific 3D printed models can be used in clinical practice for pre-operative training, improving patient safety.

## Introduction

### Medical simulation and skull base surgery

Medical simulation is a growing field, and a major objective for medical education and surgical skills acquiring in the twenty-first century. US government published a bill (Now Act of 2009 [1]) which defines medical simulation as “the use of a device, such as a mannequin, a task trainer, virtual reality, or a standardized patient, to emulate a real device, patient, or patient care situation or environment to teach therapeutic and diagnostic procedures (…) to a health care professional". The purpose of medical simulation is to improve patient safety, reduce healthcare costs and promote the development of innovative procedures. Simulation is particularly useful in skull base surgery. In this field, surgical techniques are constantly evolving thanks to the development of endoscopic transnasal approach. It requires training surgeon students to these complex techniques where there is no direct visual feedback. This implies in particular to accurately learn the nose and skull base anatomy (and to train to base surgical gestures on a complementary haptic feedback). Realistic replicas of anatomical specimens may be used to represent fine anatomical details [2] such as bone relief surgical landmarks. Pre-operative simulation of a specific and complex surgery provides a unique opportunity to employ surgical steps in order to determine the best operating strategy [3–4]. Notwithstanding raised and still evolving quality standards, surgeons performing routine endoscopic endonasal interventions are faced with minor complications in 5% and major complications in 0.5–1% [5]. In this context, simulating these interventions is essential to learning and enhances safety in the operating room. The endoscopic endonasal approach is employed in the treatment of pituitary adenomas, Rathke’s cleft cysts, skull base meningiomas, craniopharyngiomas, chordomas, olfactory neuroblastomas, and sinonasal carcinomas involving the skull base, among other pathologies.

### State of the art of skull base models

We can define two types of simulators for surgical procedures: virtual (based on environments rendered by computers) and physical (based on real objects) simulators. Virtual simulators have been developed in recent years with or without haptic feedback. The two more successful projects are the McGill Simulator for endoscopic sinus surgery simulator (MSESS) [6] and the Endoscopic Sinus Surgery Simulator (ES3) [7]. Although these simulators have shown interest in surgical training, virtual simulation requires a team of developers fully dedicated to the completion of the simulator, generating high production costs over an extended period. It seems difficult too, for physicians, to implement new anatomical or pathological conditions on these models without extra IT development. Physical simulators are easier to achieve in a reasonable period. Several physical simulators in endoscopic skull base procedures were created [8]. Foremost, low-cost simulators can be built with gelatin [9] in order to acquire base surgical gesture in endoscopic procedures. Even if the small cost is attractive ($5 USD) it does not allow trainees to accurately learn anatomy or complex surgical approaches. In the same way, some learning programs created to develop specific skills needed in skull base endoscopic surgery use a multitask model box, to acquire certain psychomotor dexterity [10]. Once again, emphasis is placed on acquiring the gesture, without trying to reproduce faithfully the human body. At the opposite, some models simulating skull base bone and soft tissues (mucosa, turbinates, vessels…) are already available. We may mention the S.I.M.O.N.T (Sinus Model Otorhino-Neuro Trainer) [11], commercialized by Pro Delphus (Olinda, Pernambuco, Brazil), and the incisive human nasal model of endoscopic surgery training by SurgTrainer (Tsukuba city, Ibaraki, Japan) [12]. The S.I.M.O.N.T. is made in resin, to simulate human bone, coated with a material called Neoderma which simulate soft tissues as mucosa. No data are available in the literature regarding properties of the materials they used. SurgTrainer model is made in plaster with replaceable parts and commercialized for $2000 USD. Although it proved its efficiency for endonasal surgical procedures training [13], mechanical properties were only evaluated by subjective experiments (i.e tactile feedback during drilling). Finally, some simpler 3D printed models could be a good compromise between controlled costs and high-fidelity. V. Waran et al. [14] published results concerning the anatomical accuracy for such a model. Unfortunately, they neither mention material they used nor quantitative data related to its mechanical properties. The approximated costs were about $7500 USD.

For physical simulation, the ideal material has to be low-cost and needs appearance, mechanical behavior and anatomical (geometric) high-fidelity. Indeed, anatomical accuracy is important for surgical learning and pre-operative surgical planning [3, 15–17]. We have to strongly consider mechanical accuracy too, because of the risk of serious injuries due to abnormal forces applied with surgical tools. It can be dangerous in clinical practice because trainees could damage important functional or vital entities (i.e. internal carotid artery, optic nerves, meninges, orbits…) and lead to patient death or serious handicap. So, the mechanical validation of material is essential for patient safety. Surprisingly, no quantitative data are available in the literature regarding the geometric accuracy or properties of the materials that are used in these physical simulators. It is a major concern as the anatomical and the haptic feedback accuracy are key-elements for the learning process [14]. Tai and al. [18] developed a 3D-printed physical simulator for endoscopic endonasal drilling for an approximate cost of $500 USD with only a subjective evaluation of haptic feedback. Recently, Alrasheed and al. [19] described the development and validation of a 3D-printed model for the training of endoscopic sinus surgery skills. Material was only chosen according to its properties provided by the manufacturers (VeroWhitePlus RGD835) without specific quantitative evaluation during simulation procedures.

## Objectives

**Fig 1:**
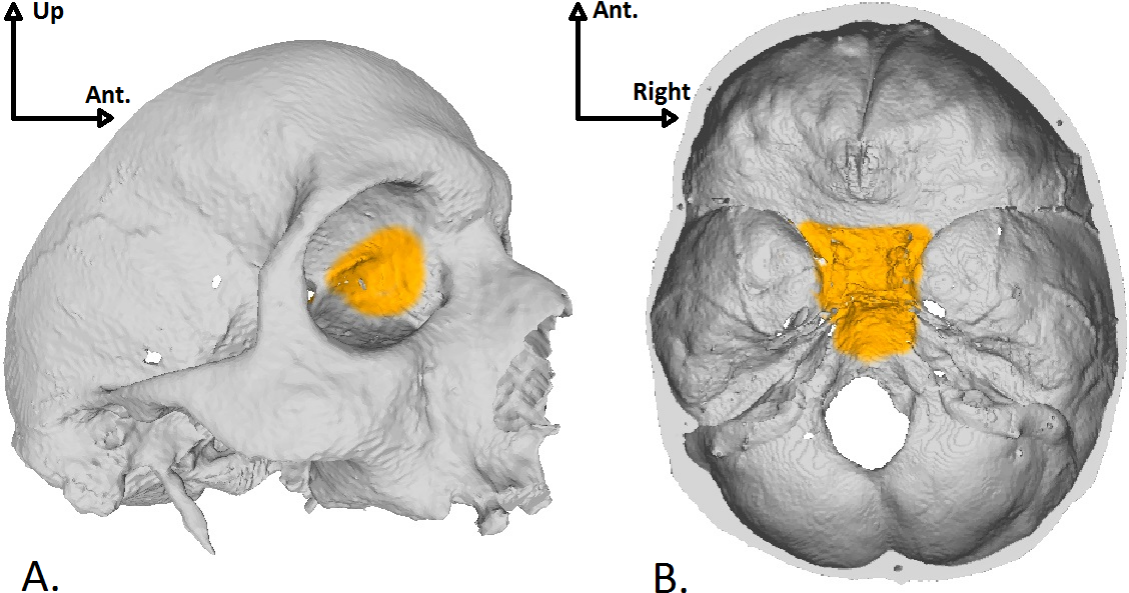
Skull base regions of interest. A: View of the left orbital lamina which is a smooth and oblong bone plate, part of the lateral surface of the ethmoid bone. B: Endocranial view of the body of the sphenoid bone.

The purpose of this work was to compare the geometric and mechanical properties of several 3D-printing consumer-grade materials with human cadaverous skull base bone to determine the best material for building a skull base model applied to endonasal surgery simulation. We aim to find a market-available material with high-fidelity concerning anatomical accuracy and mechanical behavior during common endoscopic surgical gestures. Material should have a visual aspect similar to human bone, surface properties allowing surgical tools grip, reasonable production costs and delivery time compatible with a clinical practice. We based our study on procedures used in endonasal surgery as breaking ethmoidal walls, including the orbital lamina (see Fig 1A), breaking the anterior wall of the sphenoidal sinus or drilling the clivus of the sphenoid bone to access the posterior cranial fossa (see Fig 1B).

## Materials and methods

**Fig 2:**
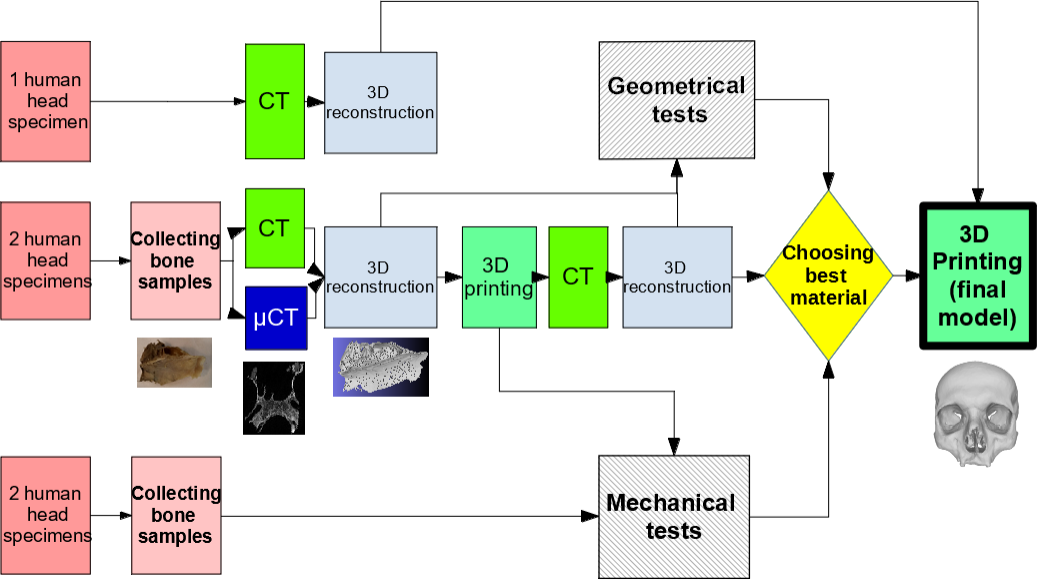
Main steps for geometric and mechanical evaluation of materials. Five human cadaverous heads were used: first one was a control head requiring medical scanner (CT) acquisition only; 2 were dissected to perform bone samples and acquired in CT and microCT scanner (μCT), then 3D-printed; the last two were only dissected to perform bone samples. 3D-printed samples were in turn acquired in CT. Two types of experiments were then realized: geometric tests on 3D computer reconstruction of bone samples acquired in CT and μCT, compared with those of 3D-printed samples; mechanical tests on bone samples and 3-D printed samples.

The main steps are summarized in Fig 2.

### Anatomical dissections

Five frozen cadaverous heads from the anatomy laboratory of Montpellier (France) were used in this study. The first one was only acquired in CT. Four frozen cadaverous heads were used as bone samples. All cadaver were donated to an institutional donation center (University of Montpellier, France), with consent obtain from the donor. Anatomical dissections have been realized according best practices from the school of medicine of Montpellier. This study is in accordance with the Helsinki Convention and the French legislation on the donation of bodies. None of the donors were from a vulnerable population. Written informed consent was obtained from the donors for anatomical and surgical studies. In this context, authors did not seek approval of specific ethic committee to approve the study. Human samples were collected from February 2016 to April 2016. Anatomical entities of interest were the orbital laminae of ethmoid bone, the anterior wall of sphenoidal sinus and the body of sphenoid bone. To the extent possible, bone samples were cleared of soft tissues. However, if this process may weaken bone structure, some tissue layers were preserved (i.e. periosteum).

### Imaging acquisition

We chose to compare the geometric accuracy of materials using 3D mesh reconstructed from computed tomography (CT) acquisitions [15]. A mesh is a virtual 3D model consisting of polygons. In this study, polygons were triangles jointed together by a vertex. These vertices were then used to perform geometrical tests. To avoid data loss due to non-optimal imaging resolution, we also compared geometric properties between CT and micro-scanner (μCT) imaging. We considered μCT as the gold standard for skull-base bone imaging because it is currently the non-destructive 3D imaging technique which provides the best accuracy/acquisition time ratio. It is mainly used and designed for industrial and small-animal imaging. μCT is able to precisely detail the fine bone walls that are not visible to the CT. Specifications of these technologies were as follows:

- CT: we used a GE Discovery CT750 HD (Chicago, Illinois, US) at the University Hospital Gui de Chauliac (Montpellier, France) with 625 μm slice thickness (z-axis) and 406 μm x- and y-axis resolution. Control-head, bone samples of 2 dissected cadaverous heads (orbital lamina, anterior wall of sphenoidal sinus, body of sphenoid bone) and 3D-printed samples were also imaged with this scanner.
- μCT: we used a Brucker RX Skyscan 1076 (Kontich, Belgium) configured for 36 μm slice thickness. We choose to secondarily decrease the resolution to 124 μm slice thickness for time consuming matter. Only bone specimens were acquired with μCT and not the materials.

### Post processing of imaging data

**Fig 3:**
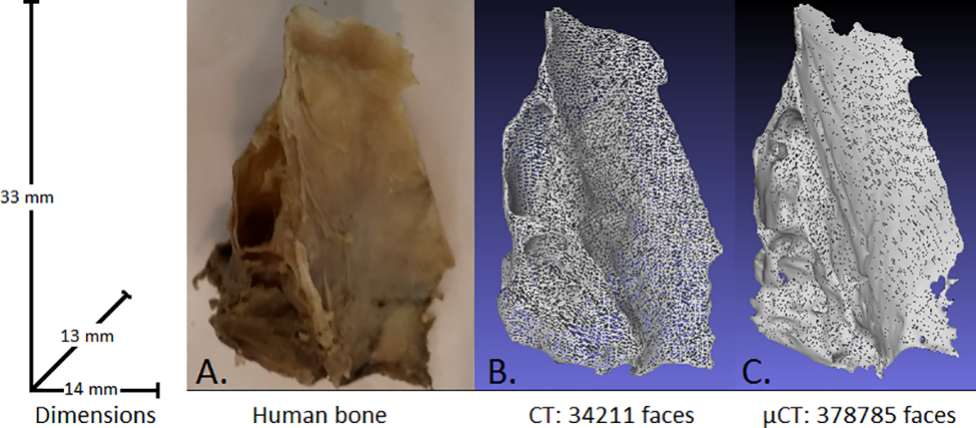
Illustration of imaging accuracy. A. Photography of an orbital lamina, B. CT-mesh, C. μCT-mesh.

According to [14], we performed post processing steps with free-software platforms. In medical image processing, segmentation means selection of voxels on slices in order to gather them in region of interest (ROI) representing an anatomical or pathological entity. We performed segmentation of skull base and sinuses bone with Medical Image Segmentation Tool (MIST, freeware, available on https://github.com/BenjaminGilles/MIST) on CT and μCT imaging to get 3D objects (Fig 3). Mucosa, nerves, vessels and other soft tissues were excluded from the segmentation. ROI were segmented using a combination of semi-automatic intensity thresholding (beyond 300 Hounsfield Units), region growing and slice-by-slice manual contouring in orthogonal tri-planar view. For thin lamellar bone, an automatic segmentation process based on region growing was corrected after by an expert with manual interaction. Then a surface reconstruction process was carried out, obtaining a 3D mesh composed of triangles which is a discrete representation of the boundary surface of the ROI. In fact, meshes are obtained from semi-automatic segmentation of CT or μCT slices. So, it is somehow a stacking of submeshes which corresponds to the segmented slices. Because of the inaccuracy in the segmented slices, the resulting mesh may be quite irregular in shape. It requires then a post-processing called smoothing which attenuates the defects and produces a good-looking mesh, allowing high 3D printing quality. Smoothing was done using Meshlab (freeware, http://meshlab.sourceforge.net/) to get a more realistic surface.

### 3D printing

3D meshes of bone samples were printed in four consumer-grade materials (Table 1): Multicolor (plaster, as used by Tai and al. [18]), resin (already used by Alrasheed and al. [19]), polyamide (PA) and polycarbonate (PC). Printers were not prototypes or specially prepared for the study. Multicolor, resin and PA were printed via Sculpteo (Villejuif, France). PC was printed at the Laboratory of Informatics, Robotics and Microelectronics (LIRMM, University of Montpellier, France). Printing paths were automatically determined for all materials.

**Table 1:** Table summarize material and printer properties as specified by manufacturers.

Material properties	Multicolor	Resin	PA	PC
**Material denomination**	Calcium sulfate hemihydrate	Opaque resin	Polyamide	Polycarbonate
**Material manufacturer**	ZP 151, 3D Systems Europe Ltd., Hemel Hempstead, UK	VeroWhite Plus RGD835, Stratasys, Eden Prairie, Minnesota, US	EOS, Krailling, Deutschland	Stratasys, Eden Prairie, Minnesota, US
**3D printer**	3D Zprinter 650, ZCorp, 3D Systems	Object30 Pro 3D printer, Stratasys	EOS formiga printer (from P100 to P730 models)	Fortus 400mc printer, Stratasys
**3D printer accuracy (μm)**	400	200	150	125
**Printing technology**	Binder jetting	Photopolymerization	Selective laser sintering	Fused deposition modeling
**Young's Modulus (N/mm**^**2**^**)**	6405	2000–3000	1700	1956
**Yield strenght (N/mm**^**2**^**)**	9	50–60	45	30–40
**Melting point (°C)**	1459	N/A	172	N/A
**Glass transition temperature (°C)**	N/A	52	N/A	161

### Geometric comparison

Geometric tests were based on medical imaging comparison between materials and bone samples. In order to evaluate the 3D printing materials geometrical accuracy, we chose to compare the shapes of the 3D-printed samples with respect to the one of original bone. Bone and materials samples were imaged with CT and segmented to build meshes. Then, we performed a global mesh comparison between bone and materials by computing the average distance between all the vertexes of the two registered meshes (rigid register) to assess global accuracy. To do this, meshes were superimposed at best to remove the differences of position and orientation. Then, it allows the comparison of the resulting geometry differences by measuring the average distance between vertices. It can be visualized as a color map of distances. This measurement quantifies the global deformation of the samples with respect to the standard shape which is given by the original bone. This step was done using CloudCompare freeware (http://www.danielgm.net/cc/). Mesh accuracy comparison was performed between:

-    (1) smoothed meshes of bone and materials in CT imaging,
-    (2) non-smoothed meshes of bone and smoothed meshes of bone (to evaluate the effect of smoothing steps on geometry accuracy),
-    (3) smoothed meshes of bone in CT and smoothed meshes of bone in μCT (to evaluate the accuracy of medical scanner imaging to build models with 3D prototyping technologies).

Nevertheless, this global evaluation of geometry does not emphasize any local deformations. Finally, we compared distances between couples of anatomical point of interest selected by an expert (the upper and anterior point of posterior clinoid process of sphenoid bone) in bone and materials to assess local accuracy.

### Mechanical comparison

To evaluate haptic forces, experiments used real surgical tools to measure forces applied on materials during surgical procedures. The two main procedures in endoscopic sinus surgery are osteotomy (breaking thin walls) and drilling. Osteotomy may be performed with a rigid suction tip. Then, experiments evaluated forces applied by the suction tip on materials during osteotomy simulation (breaking materials). Drilling was performed with powered devices designed for endoscopic sinus surgery. Forces applied on materials during drilling were measured with a force sensor fixed between the drill and surgeon’s hand. All mechanical comparison of materials was performed with these two tests on excised pieces of bones and on materials. We calculated the average force needed to break thin walls with a surgical suction tip used in endonasal surgery. A force sensor (F/T sensor Mini45, ATI industrial automation, Apex, NC, USA) was used with an upper limit set to 200 N (tests were aborted if the material was not broken at 200 N). An additional mechanical test was performed on thicker samples part (i.e. clivus of sphenoid bone) with a surgical drill (4 mm diamond bur and 12000 rpm motor, Karl Storz, Tuttlingen, Germany), the same force sensor and a position tracker (easyTrack500, Atracsys, Zug, Swiss). Positions and forces applied on the bur during a 5 second drilling were measured. Then, we estimated the energy spent and reported instantaneous forces during a 6mm depth drill.

All material samples were printed in triplicate to repeat measurements and calculate the average forces and standard deviations.

## Results

### Geometric comparison

**Fig 4:**
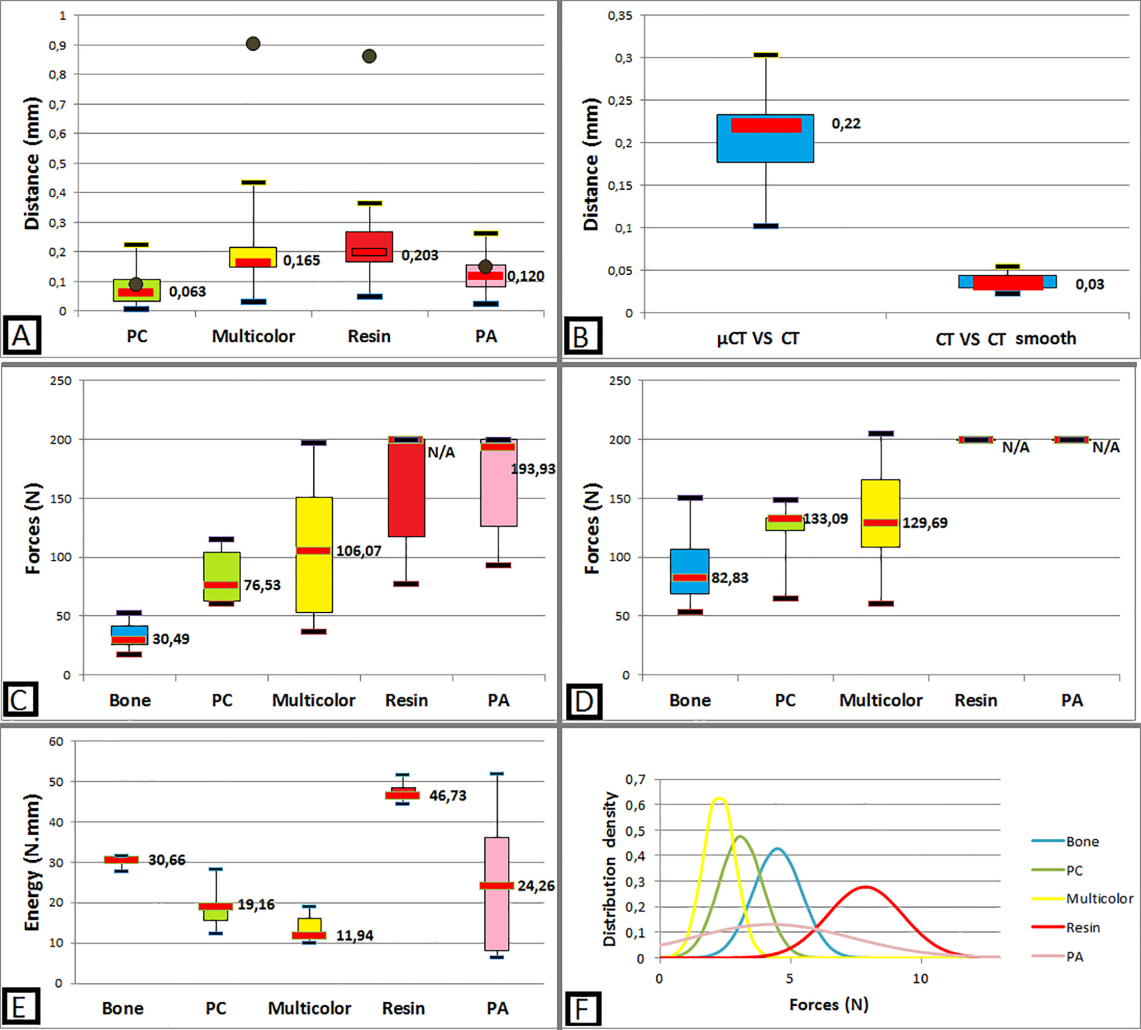
Main mechanical and geometric tests results. A: Absolute distance between material mesh compared to bone mesh (boxplot). Distance error between material and bone landmarks (brown points). B: Bias evaluation: accuracy of CT compared with μCT and smoothing steps. C: Forces required to break thin walls (orbital lamina). D: Forces required to break thin walls (anterior wall of the sphenoidal sinus). E: Comparison of energy spent in a 6mm depth drilling on cadaver bone and materials. F: Repartition of instantaneous forces applied on materials during a 48mm depth cumulated drilling.

As shown in Fig 4A and 4B, PC and PA had the highest local and global accuracy. Proportionally, a difference of 0.083mm between bone and PC surface represents approximatively a global error (distance between surfaces/sample dimensions) of 0.36% (for a 23mm diameter sample). All materials were satisfying on global geometric accuracy. According to the same process, the landmarks analysis showed that resin and Multicolor are not enough locally accurate, with an average error of 0.86 and 0.90mm for a real distance of 12.5mm. The geometrical accuracy of PA and PC was 6 and 7 times greater than in resin and Multicolor. PC and PA local and global accuracy are sufficient to allow safety training without risk of aberrant injury of vital structures (i.e. internal carotid artery or optic nerves). The average distance between meshes obtained with CT and μCT was 0.207mm (SD 0.65mm). Geometric difference between non-smoothed and smoothed meshes was very low and acceptable.

### Mechanical comparison

Human cadaver bone was more fragile than materials (see Fig 4C and 4D). Applied forces were 1.6 to 2.5 times higher in PC, and 1.6 to 3.5 times higher in Multicolor. For resin and PA, forces exceeded 200N which is not applicable on patients. Energy spent during a 6mm depth drilling (Fig 4E) was respectively 1.6 and 2.6 times higher on bone than on PC and Multicolor. Instantaneous forces when drilling followed a normal distribution for all materials except PA which was melted (Fig 4F).

### Macroscopic aspect of materials

**Fig 5:**
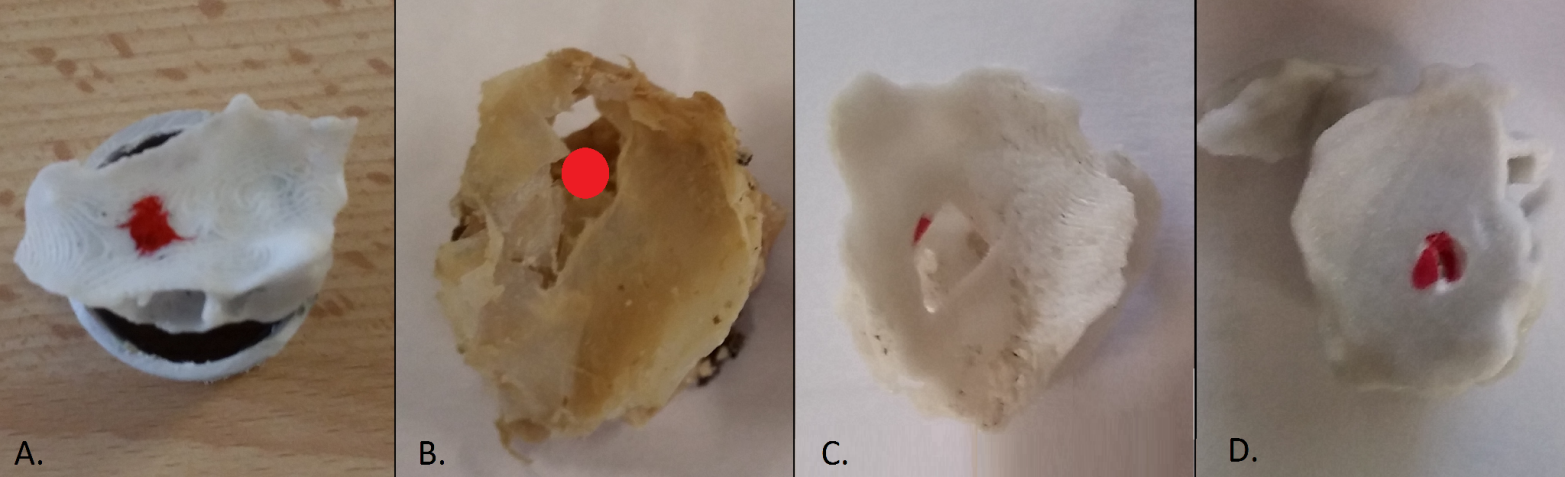
Macroscopic aspect of broken materials (orbital lamina). Red point represents the pressure point. **A**: Sample positioned on a circular support for the breaking test. **B:** Cadaver bone, **C**: PC: the fracture mode is similar to the cadaver bone, **D**: Multicolor: the fracture line is distant to the pressure point.

All materials had a color compatible with bone aspect (shades of white or grey), except PA which was too white and could cause difficulties in endoscopic procedures with light reflection. During breaking tests, PA undergone visible plastic deformation, Multicolor and resin undergone a fracture line located remotely from the pressure point while PC had a mode of fracture similar to the human bone (localized at the pressure point without aberrant extension, see Fig 5). During drilling, PA was melted, as the superficial shape of Multicolor (coated with cyanoacrylate), and became adhesive to the bur. Drilling resin produced a high-pitched noise. Multicolor and PC drilling subjective perception was approximately same as bone.

## Discussion

### Methodology and bias control

We consider PC as a good compromise between high anatomical and mechanical accuracy, visual and surface appearance, acceptable delivery time and costs production (Table 2). For all materials, the time between purchase and delivery did not exceed a week.

**Table 2:** Table summarize material properties regarding initial specifications.

	PC	Multicolor	Resin	PA
**Visual aspect**	√	√	√	⨂ (too white)
**Surface roughness**	√	√	⨂ (too smooth)	√
**Mechanical properties**	√	⨂	⨂	⨂
**Geometrical accuracy**	√	⨂	⨂	√
**Reprodutibility**	√	√	√	√
**Costs**	155$ (excl. Tax)	275 $	880 $	298 $
**Production time**	√ (3 days)	√ (3 days)	√ (4–5 days)	√ (2–5 days)

√: Material complies with requirement; ⨂: Material is not compliant with requirement. Delivery time was about two open days.

In order to evaluate the geometrical accuracy of the 3D-printed materials, we chose to compare the shape of 3D-printed samples with the original bone. A direct comparison of CT image is difficult for several reasons. First, a direct CT versus μCT comparison has to take into account the large difference in resolution acquisition. There were 20 times more slices with μCT imaging than CT for the same sample. A way could be to undersample the μCT image but then, the procedure will be different that between two CT images. Using meshes in all cases allows a standard procedure. Secondly, meshes are easier to visualize, especially representing distances. In this case, to get a distance map, vertices were colored with respect to the distance value in order. It allows then to know if a systematic error was done (i.e: are the concave surfaces systematically filled?). Finally, 3D printing technologies require mesh inputs to enable printing. It seems then interesting to be able to compare data which are the closes to the printer inputs.

The 3D superposition of meshes makes it possible to compare their geometry, calculate and visualize a map of distance between them. This parameter emphasizes the global deformation with respect to the standard. To evaluate more localized deformation, we compared the distance between two anatomical landmarks selected by an expert. A variation of this distance in material samples compared to the bone should lead to local deformation and inaccuracy. It could have serious consequences if such inaccuracies occur near vital structures. As an example, in transsphenoidal surgery, if the intersinus septum was attached to the internal carotid artery, it can lead to injury when removal. On the opposite, if the septum was attached a few millimeters farther, the risk is considerably decreased.

Geometric evaluation was based on CT segmentation and may be too much imprecise to create reliable models. Furthermore, the consequences of mesh smoothing steps on the geometry were unknown. These steps were empirical and visually controlled by the operator. Mesh comparison before and after smoothing showed an average difference of 0.037mm (SD 0.11mm). We can consider that the impact of smoothing on the geometry is negligible. In order to know if medical tomography was sufficient to acquire all the fine details of objects, we compared meshes obtained with CT and μCT. The major interest of μCT is to be able to precisely detail the fine bone walls that are not visible to the CT. Still, data acquisition time was important using the Bruker 1076 device (one hour for each bone sample in our study). X-ray dose levels received by the sample were high, and a human body would not fit in most conventional industrial X-ray micro-tomographs. Thus, μCT is not used in medical practice but it is considered as a reference in terms of resolution for 3D imaging of bones. The average distance between meshes was 0.207mm (SD 0.065mm) and the voxel size of the CT was 0.406x0.406x0.625mm. The difference between meshes obtained with CT and μCT was less than half a voxel, which is the usual segmentation bias due to partial volume effect [20]. The choice of tested materials was initially determined by their accessibility to general public (consumer-grade materials), with industrial-designed 3D printers without any special preparation, in order to permit each surgeon to build specific model in his center.

Sources of errors in 3D printing are multiples [21], depending on 3D printers and materials. Some materials need post printing steps that could damage samples as cleaning. Multicolor needs manual cleaning and gluing with cyanoacrylate then varnishing. PA needs to be sandblasted to remove the unused fine powder. Resin needs a pressurized water jet to remove any excess liquid. PC support matrix has to been dissolved in an aqueous solution with ultrasounds. The use of a 3D printer service business can induce printing limitations due to the need for profitability. For example, for resin and Multicolor, printing technologies allow a reusing of 3D printing material which is not needed in the final object (unused liquid polymer for resin, and unused powder for Multicolor). It is important for Multicolor and resin that there is no risk of broken part during the printing process; otherwise it could damage the printer. So, the printing company thickens some parts of the input meshes on its own initiative. This may create some distortions and affect the final geometry.

### Mechanical characterization

Human skull bone mechanical properties have already been studied in several studies. There is a large variation in the recorded mechanical properties of cranial bone, relate to chosen testing parameters: how tissue is preserved prior to testing, the type of loading used and the testing speed [22–23]. However, compression, tension or bending properties do not represent those which are useful for surgical simulation. Waridel and al. [24] measured the mechanical properties of the sphenoid and ethmoid sinus bones, but their results are limited to very specific anatomical regions. In our study, bone resistance at break is probably underestimated due to ex vivo tests on little size samples and multiple manipulations (anatomical dissection and sampling, CT, μCT) with repeated freezing or defrosting periods. According to White and al. [25], cadaver tissue may underestimate the mean force required in osteotomy of living ethmoid sinus lamellae by a factor of 1,5 times.

Classic evaluation of mechanical behavior of material (as Young’s modulus) would not be representative for physicians. Indeed, such experiments do not correspond with surgical practice. Basic tests include generally tension, compression or hardness tests on calibrated samples of materials. For bone evaluation, it was impossible to obtain calibrated samples, because of geometry (anatomy). It seemed difficult to compare material and bone properties without calibrated tests. That is the reason why mechanical behavior of materials was characterized with surgical tools used in operative room.

There are two main procedures in endoscopic skull base surgery. First, thin bone walls can be broken with non-blunt surgical tools as surgical suction tips (osteotomy): surgeon have to apply a normal force on bone surface to break it. Thus, measurements represent the resistance at break of bone and materials. During surgery, operator use this procedure distance to important anatomical entities. So, material just has to be able to break without aberrant fracture. A force sensor was placed between the suction tip and surgeon’s hand to measure forces applied by the suction tip on material.

Secondly, surgeon could perform a surgical drilling on thick bone parts to access a pathological process. A force sensor was placed between the drill and surgeon’s hand. We tried to model forces applied on the bur and energy spent for a one directional calibrated drilling at constant speed. These tests do not represent the exact surgical procedure which consists in a surface drilling with 3-axis bur displacement. Modelling this procedure was not feasible because of the difficulty to measure forces applied on a wider area. The two mechanical tests performed in this study are inspired by stress tests: they represent tolerance of materials to maximum bending (force applied for breaking thin walls) or drilling. These tests directly represent surgical gesture and haptic feedback which is essential to evaluate in surgical simulation.

A third procedure consists in bone removal with forceps. Radley et al. [26] described a method to measure forces applied on through-cutting surgical rongeurs. But devices and sensors used to characterize these forces are not easy to use, and it does not consider the accuracy of fracture line induced by the forceps. Indeed, for a same applied force on forceps in bone and material, if the fracture line extends widely over forceps jaws on material surface, the surgical accuracy might be dangerously decreased. As shown in Fig 5, PC had a mode of fracture closest to the cadaver skull base bone. Applied forces at break were higher than on bone, but we can consider bone cadaver strength is weaker than in vivo, due to conservation methods (multiple freezing and defrosting for experiments) and a possible embrittlement of samples inherent in collecting technique on cadaver which is not at all feasible in a living patient. Furthermore, leaving bone is surrounded by conjunctive tissues which reinforce resistance at break. Orthopedic studies suggest the importance of soft tissues properties to prevent bone fractures. Thus, for hip fractures, energy absorption in soft tissues may account for up to 75% of the energy in fall [27]. Bouxsein and al. [28] showed that qualitative and quantitative properties of hip soft tissues influence fracture risk during a sideway falls.

Das et al. [29] also used PC to simulate skull bone for ballistic projectile evaluation. They consider PC to be too ductile for this application, which confirms the need to carry out mechanical tests specific to the chosen application. Finally, drilling procedures are used close to important anatomical entities. Concerning forces applied during drilling, PC was slightly less resistant than human cadaver bone. Consequently, this difference could not be responsible of patient serious injuries, because surgeon trainee will apply fewer forces when drilling on a patient in the operative room.

### Costs

The approximate cost for a model is $155 USD of PC material [Table 2]. A model can be used bilaterally, allowing 2 trainings procedures or one training for 2 trainees. Learning curves in functional endoscopic sinus surgery depend on surgical procedures. According to Fried and al. [30], for basic procedures such as middle meatotomy, students need 6 trials to equal performance of residents and attending.

### Limits

This model do not includes soft tissues as mucosa, perichondrium or periosteum which play an important role in dissection time (and to a lesser extent in mechanical resistance of bone). It limits visual realism. Some surgical procedures which need surgical dissection may not be simulated yet. Furthermore, there are no vascular or nervous structures visible in surgery. However, it seemed essential to find a material whose mechanical properties were close to the human bone. We focused on anatomical fidelity (by geometrical comparison) and haptic feedback fidelity (by mechanical tests) as they are fundamental for clinical practice. These properties are the first elements of validation for training procedures. Indeed, it can be used for basic skills acquisition (and endoscopic anatomy learning) for students or for surgical planning. The major advantage is the personalization of 3D printed models to train on a wide range of anatomies while being in a reasonable cost range.

Full segmentation time currently required about ten hours, which is a limit to products and uses these models. Segmentation automation of skull base bone is a major concern for our team. Various solutions exist but remain partial and cannot be generalized to all the bone structures of the skull base [31–32].

### Perspectives

**Fig 6:**
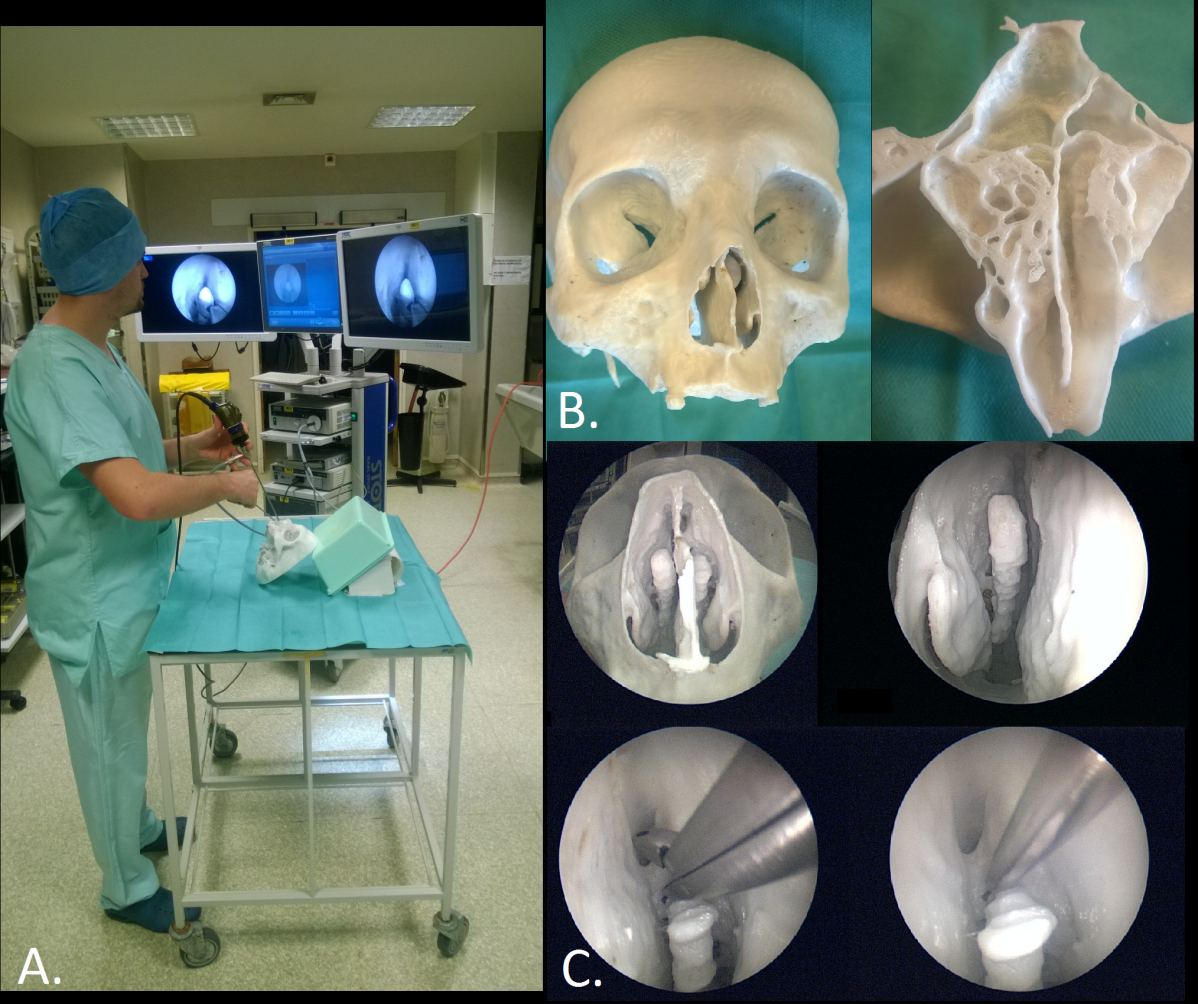
Surgical training. A. View of the installation for surgical training. B. View of the entire 3D-printed model (on the left) and internal details of ethmoidal and sphenoidal sinuses (on the right). C. Endoscopic views of the model and training procedures: resection of ethmoidal cells with a rongeur and breaking walls with a suction tip.

Polycarbonate (PC) is able to simulate the bone of a cadaver skull base to learn anatomy and perform surgical training endoscopic procedures with geometric and mechanical reliability. This simulator can be used by both neurosurgeons and ENT surgeons to perform endoscopic endonasal skull base surgery. It can also be used in non-endoscopic neurosurgery training to acquire drilling skills, or in common endonasal procedures such as lower or middle meatotomy training. This precise quantitative evaluation of geometric and mechanical properties of PC permits to extrapolate these results and allows the creation of patient-specific models for pre-operative training (see Fig 6) and evaluation, surgical planning in selected cases or development of new surgical approaches, medical device tests or robotic-assisted surgery evaluation. As the present study, a previous work on mechanical properties of mucosa will be carried out. Future works are needed to integrate soft tissues substituting materials and determine the contribution of such models to surgical education (face, content and construct validity of simulator).

## Conclusions

3D printing technologies are more and more used in medical and surgical simulation, because of their ability to build a model and a playful approach. Most of skull base models already used these technologies, but none assessed geometric and mechanical accuracy compared with cadaver bone. To the best of our knowledge, our work is the first attempt to quantitatively validate the use of printed material for skull base surgery simulation. We propose polycarbonate (PC) as a good material to simulate skull base bone, based on quantitative characterization of its properties in surgical condition. Thanks to short lead times and reasonable production costs, patient-specific 3D printed models can be used in clinical practice for anatomical education and pre-operative training, improving patient safety.

## References

[1] Forbes J. H.R.855—111th Congress (2009–2010): Enhancing Safety in Medicine Utilizing Leading Advanced Simulation Technologies to Improve Outcomes Now Act of 2009 [Internet]. 2009 [cited 2017 Aug 10]. Available from: https://www.congress.gov/bill/111th-congress/house-bill/855

[2] McMenamin PG, Quayle MR, McHenry CR, Adams JW. The production of anatomical teaching resources using three-dimensional (3D) printing technology. Anat Sci Educ. 2014 12;7(6):479–86. doi: 10.1002/ase.1475 24976019 10.1002/ase.1475

[3] Mavili M, Canter H, Saglam-Aydinatay B, Kamaci S, Kocadereli I. 2007 Use of three-dimensional medical modeling methods for precise planning of orthognathic surgery. J Craniofac Surg 18:740–747. doi: 10.1097/scs.0b013e318069014f 17667659 10.1097/scs.0b013e318069014f

[4] Rengier F, Mehndiratta A, von Tengg-Kobligk H, Zechmann CM, Unterhinninghofen R, Kauczor H-U, et al 3D printing based on imaging data: review of medical applications. Int J Comput Assist Radiol Surg. 2010 7;5(4):335–41. doi: 10.1007/s11548-010-0476-x 20467825 10.1007/s11548-010-0476-x

[5] Hosemann W, Draf C. Danger points, complications and medico-legal aspects in endoscopic sinus surgery. GMS Curr Top Otorhinolaryngol Head Neck Surg. 2013 12 13;12:Doc06 doi: 10.3205/cto000098 24403974 10.3205/cto000098 PMC3884541

[6] Varshney R, Frenkiel S, Nguyen LH, Young M, Del Maestro R, Zeitouni A, et al The McGill simulator for endoscopic sinus surgery (MSESS): a validation study. Journal of Otolaryngology—Head & Neck Surgery. 2014;43:40. 25927463 10.1186/s40463-014-0040-8 PMC4210497

[7] Solyar A, Cuellar H, Sadoughi B, Olson TR, Fried MP. Endoscopic Sinus Surgery Simulator as a teaching tool for anatomy education. Am J Surg. 2008 7;196(1):120–4. doi: 10.1016/j.amjsurg.2007.06.026 18374890 10.1016/j.amjsurg.2007.06.026

[8] Javia L, Deutsch ES. A systematic review of simulators in otolaryngology. Otolaryngol Head Neck Surg. 2012 12;147(6):999–1011. doi: 10.1177/0194599812462007 23014997 10.1177/0194599812462007

[9] Malekzadeh S, Wilson B. A Novel Low-Cost High-Fidelity Sinus Surgery Task Trainer. Otolaryngology—Head and Neck Surgery. 2010 8 1;143(2 suppl):P267–P267. 10.1177/0194599811413373 21690274

[10] Sanromán-Álvarez P, Simal-Julián JA, Miranda-Lloret Pablo null. Multitask box trainer for endoscopic endonasal skull base surgery: ENDOtrainer. World Neurosurg. 2017 2 20. 10.1016/j.wneu.2017.02.008 28232211

[11] Nogueira JF, Stamm AC, Lyra M, Balieiro FO, Leão FS. Building a real endoscopic sinus and skull-base surgery simulator. Otolaryngol Head Neck Surg. 2008 11;139(5):727–8. doi: 10.1016/j.otohns.2008.07.017 18984272 10.1016/j.otohns.2008.07.017

[12] Yamashita J, Morikawa O, Hashimoto R, Fukui Y, Yamauchi Y, McChimaru M, et al. Manikin and method of manufacturing the same [Internet]. US20070020598 A1, 2007 [cited 2016 Sep 12]. Available from: http://www.google.tl/patents/US20070020598.

[13] Chan HHL, Siewerdsen JH, Vescan A, Daly MJ, Prisman E, Irish JC. 3D Rapid Prototyping for Otolaryngology—Head and Neck Surgery: Applications in Image-Guidance, Surgical Simulation and Patient-Specific Modeling. PLoS One [Internet]. 2015 9 2 [cited 2016 Nov 16];10(9). 10.1371/journal.pone.0136370 PMC4557980 26331717

[14] Waran V, Menon R, Pancharatnam D, Rathinam AK, Balakrishnan YK, Tung TS, et al The creation and verification of cranial models using three-dimensional rapid prototyping technology in field of transnasal sphenoid endoscopy. Am J Rhinol Allergy. 2012 10;26(5):e132–6. doi: 10.2500/ajra.2012.26.3808 23168144 10.2500/ajra.2012.26.3808

[15] Torres K, Staśkiewicz G, Śnieżyński M, Drop A, Maciejewski R. Application of rapid prototyping techniques for modelling of anatomical structures in medical training and education. Folia Morphol (Warsz). 2011 2;70(1):1–4. 21604245

[16] Chae MP, Rozen WM, McMenamin PG, Findlay MW, Spychal RT, Hunter-Smith DJ. Emerging Applications of Bedside 3D Printing in Plastic Surgery. Front Surg. 2015;2:25 doi: 10.3389/fsurg.2015.00025 26137465 10.3389/fsurg.2015.00025 PMC4468745

[17] Sander IM, McGoldrick MT, Helms MN, Betts A, van Avermaete A, Owers E, et al Three-dimensional printing of X-ray computed tomography datasets with multiple materials using open-source data processing. Anat Sci Educ. 2017 2 23. 10.1002/ase.1682 28231405

[18] Tai BL, Wang AC, Joseph JR, Wang PI, Sullivan SE, McKean EL, et al A physical simulator for endoscopic endonasal drilling techniques: technical note. J Neurosurg. 2016 3;124(3):811–6. doi: 10.3171/2015.3.JNS1552 26339850 10.3171/2015.3.JNS1552

[19] Alrasheed AS, Nguyen LHP, Mongeau L, Funnell WRJ, Tewfik MA. Development and validation of a 3D-printed model of the ostiomeatal complex and frontal sinus for endoscopic sinus surgery training. Int Forum Allergy Rhinol. 2017 6 14; 10.1002/alr.21960 28614638

[20] Kessler RM, Ellis JR, Eden M. 1984 Analysis of emission tomographic scan data: limitations imposed by resolution and background, J. Comput. Assist. Tomogr. (3):514–522. 6609942 10.1097/00004728-198406000-00028

[21] Hernandez DD. Factors Affecting Dimensional Precision of Consumer 3D Printing. IJAAA. 2015 9 21; 2(4).

[22] Falland-Cheung L, Waddell JN, Chun Li K, Tong D, Brunton P. Investigation of the elastic modulus, tensile and flexural strength of five skull simulant materials for impact testing of a forensic skin/skull/brain model. J Mech Behav Biomed Mater. 2017 2 20;68:303–7. doi: 10.1016/j.jmbbm.2017.02.023 28236695 10.1016/j.jmbbm.2017.02.023

[23] Motherway JA, Verschueren P, Perre GV der, Sloten JV, Gilchrist MD. The mechanical properties of cranial bone: The effect of loading rate and cranial sampling position. Journal of Biomechanics. 2009 9 18;42(13):2129–35. doi: 10.1016/j.jbiomech.2009.05.030 19640538 10.1016/j.jbiomech.2009.05.030

[24] Waridel F, Monnier P, Agrifoglio A. Evaluation of the bone resistance of the sphenoid and ethmoid sinuses. Laryngoscope. 1997 12;107(12 Pt 1):1667–70. 9396684 10.1097/00005537-199712000-00017

[25] Joice P, Ross PD, Wang D, Abel EW, White PS. Measurement of osteotomy force during endoscopic sinus surgery. Allergy Rhinol (Providence). 2012;3(2):e61–5. 23342291 10.2500/ar.2012.3.0032 PMC3548610

[26] Radley GJ, Sama A, Watson J, Harris RA. Characterization, quantification, and replication of human sinus bone for surgery simulation phantoms. Proc Inst Mech Eng H. 2009 10;223(7):875–87. doi: 10.1243/09544119JEIM577 19908426 10.1243/09544119JEIM577

[27] Bone Health and Osteoporosis: A Report of the Surgeon General. Office of the Surgeon General (US). Rockville (MD): Office of the Surgeon General (US); 2004. 20945569

[28] Bouxsein ML, Szulc P, Munoz F, Thrall E, Sornay-Rendu E, Delmas PD. Contribution of trochanteric soft tissues to fall force estimates, the factor of risk, and prediction of hip fracture risk. J Bone Miner Res. 2007 6;22(6):825–31. doi: 10.1359/jbmr.070309 17352651 10.1359/jbmr.070309

[29] Das R, Collins A, Verma A, Fernandez J, Taylor M. Evaluating Simulant Materials for Understanding Cranial Backspatter from a Ballistic Projectile. J Forensic Sci. 2015 5 1;60(3):627–37. doi: 10.1111/1556-4029.12701 25739515 10.1111/1556-4029.12701

[30] Fried M, Satava R, Weghorst S, Gallagher A, Sasaki C, Ross D, et al Identifying and reducing errors with surgical simulation. Qual Saf Health Care. 2004 10;13(Suppl 1):i19–26. 15465950 10.1136/qshc.2004.009969 PMC1765795

[31] Alsufyani NA, Hess A, Noga M, Ray N, Al-Saleh MAQ, Lagravère MO, et al New algorithm for semiautomatic segmentation of nasal cavity and pharyngeal airway in comparison with manual segmentation using cone-beam computed tomography. Am J Orthod Dentofacial Orthop. 2016 10;150(4):703–12. doi: 10.1016/j.ajodo.2016.06.024 27692428 10.1016/j.ajodo.2016.06.024

[32] Bui NL, Ong SH, Foong KWC. Automatic segmentation of the nasal cavity and paranasal sinuses from cone-beam CT images. Int J Comput Assist Radiol Surg. 2015 8;10(8):1269–77. doi: 10.1007/s11548-014-1134-5 25503593 10.1007/s11548-014-1134-5

